# Management of a swallowed denture: our experience with 34 patients

**DOI:** 10.3205/000297

**Published:** 2021-08-06

**Authors:** Vaishnevy Ganesh, Sara Drever, Joshua Agilinko, Vamsidhar Vallamkondu, Samit Majumdar, Muhammad Shakeel

**Affiliations:** 1 Department of Otolaryngology – Head and Neck Surgery, Aberdeen Royal Infirmary, Aberdeen, United Kingdom; 2 Department of Otolaryngology – Head and Neck Surgery, Ninewells Hospital, Dundee, United Kingdom

**Keywords:** swallowed dentures, management

## Abstract

**Background:** Swallowed dentures can present with upper aerodigestive tract obstruction needing urgent intervention. Removing such an ingested denture can prove challenging and needs careful planning.

**Aim:** To share our experience of managing patients with a swallowed denture focusing on the practical aspects of denture removal along with relevant literature review. We aim to raise a public health message on the safety aspect of usage of dentures.

**Subjects and methods:** A retrospective analysis of the patients managed by our team in the ENT department at two hospitals in Scotland, over 10 years (2009–2019), who were found to have swallowed a denture. Data on demographics, clinical history, examination findings and management of patients were collected and analysed using Microsoft Excel.

**Results:** A total of 34 patients were admitted with a swallowed denture, of which 24 (71%) were male and 10 (29%) were female. The mean age was 60 years (range 17–83). Of the 34 patients, 2 had a feeling of something stuck in the throat but were able to eat and drink; the rest of the patients complained about dysphagia and pain in the throat, with 2 patients also showing signs of respiratory distress. Twenty-four (71%) patients required denture removal under general anaesthetic in the theatre; 20 (59%) by rigid oesophagoscopy, 1 with tracheostomy (3%), 1 with (3%) laparoscopy and gastrostomy, and 2 (6%) with external neck exploration. Seven (20%) patients were taken to the theatre and the denture was removed with Magill forceps under light sedation using intubating laryngoscope or video laryngoscope. In 1 patient (3%), the denture material was successfully removed under flexible pharyngolaryngoscopy guidance in the clinic without sedation. The final 2 (6%) patients were reassured as no foreign body was seen on flexible laryngoscopy.

**Conclusion:** In the absence of a clear evidence of denture ingestion, a detailed history and examination are needed to identify this serious pathology. Once confirmed, the ingested denture should be removed as soon as possible to minimize the risk of serious complications.

## Zusammenfassung

**Hintergrund:** Verschluckte Zahnprothesen können eine Obstruktion des oberen Aerodigestivtrakts verursachen, die dringend einen Eingriff erfordert. Die Entfernung einer solchen verschluckten Prothese kann sich als schwierig erweisen und erfordert eine sorgfältige Planung.

**Ziel:** Wir möchten unsere Erfahrungen bei der Behandlung von Patienten mit verschluckter Prothese teilen, wobei wir uns auf die praktischen Aspekte der Prothesenentfernung konzentrieren und die relevante Literatur besprechen. Unser Ziel ist es, die Öffentlichkeit über den Sicherheitsaspekt bei der Verwendung von Prothesen aufzuklären.

**Themen und Methoden:** Es handelt sich um eine retrospektive Analyse der Patienten, die von unserem Team in der HNO-Abteilung zweier Krankenhäuser in Schottland über einen Zeitraum von 10 Jahren behandelt wurden (2009–2019) und bei denen festgestellt wurde, dass sie eine Prothese verschluckt hatten. Es wurden Daten zur Demografie, zur klinischen Vorgeschichte, zu Untersuchungsergebnissen und zum Management der Patienten gesammelt und mit Microsoft Excel analysiert.

**Ergebnisse:** Insgesamt wurden 34 Patienten mit einer verschluckten Prothese aufgenommen, von denen 24 (71%) männlich und 10 (29%) weiblich waren. Das Durchschnittsalter betrug 60 Jahre (Bereich 17–83). Von den 34 Patienten hatten 2 das Gefühl, dass etwas im Hals stecken geblieben war, sie konnten aber essen und trinken. Der Rest der Patienten klagte über Dysphagie und Schmerzen im Hals, wobei 2 Patienten auch Anzeichen von Atemnot zeigten. Vierundzwanzig (71%) Patienten benötigten eine Prothesenentfernung unter Vollnarkose im OP; 20 (59%) durch starre Ösophagoskopie, 1 mit Tracheotomie (3%), 1 mit (3%) Laparoskopie und Gastrostomie und 2 (6%) mit externer Halsexploration. Sieben (20%) Patienten wurden in den OP gebracht, um dort die Prothese mit einer Magill-Zange unter leichter Sedierung und Verwendung eines Intubationslaryngoskops oder Videolaryngoskops zu entfernen. Bei einem Patienten (3%) wurde das Prothesenmaterial in der Klinik unter flexibler pharyngolaryngoskopischer Führung ohne Sedierung erfolgreich entfernt. Die letzten 2 (6%) Patienten konnten beruhigt werden, da bei der flexiblen Laryngoskopie kein Fremdkörper zu sehen war.

**Schlussfolgerung:** In Ermangelung eindeutiger Hinweise auf eine verschluckte Prothese sind eine detaillierte Anamnese und Untersuchung erforderlich, um diese schwerwiegende Pathologie zu identifizieren. Sobald diese bestätigt ist, sollte die verschluckte Prothese so schnell wie möglich entfernt werden, um das Risiko schwerwiegender Komplikationen zu minimieren.

## Background

Dentures are commonplace amongst the UK population with one in five people wearing them [1]. The use of dentures is particularly prevalent in the older population; they serve to provide both functional and aesthetic benefits [2]. However, the wearing of dentures is not without risk. Possible complications from dentures include gum and oral mucosa irritation, ulceration, denture stomatitis and fractured artificial teeth [3]. There is also the risk of inhaling or swallowing the dentures, which can lead to airway obstruction, oesophageal perforation, bowel obstruction and bowel perforation; and long-standing denture impaction can result in serious complications including trachea-oesophageal fistula and neck abscess [4], [5], [6]. Among the foreign bodies removed from the upper aerodigestive tract, dentures are uncommonly reported in the published literature [7]. It is important to understand that a lost denture could be sitting within the upper aerodigestive tract [8]. The general principle when dealing with a foreign body in the upper aerodigestive tract is to arrange endoscopic removal of the foreign body as soon as possible [9].

## Aim

The aim is to share our experience of managing patients with a swallowed denture, focusing on the practical aspects of denture removal along with a relevant literature review. We aim to raise a public health message on the safety aspect of usage of dentures.

## Subjects and methods

This is a retrospective analysis of the patients managed by our team over 10 years (2009–2019) who were found to have swallowed a denture. All other patients presenting with other causes of acute upper airway obstruction and mucosal irritation secondary to other causes were excluded.

Data were collected on demographics, co-morbidities, clinical history, examination findings, management and subsequent care the patients needed. Microsoft Excel was used for data collection and analysis.

## Results

A total of 34 patients were identified. There were 24 male and 10 female patients with an average age of 60 years (range 17–83).

Twenty-nine patients were referred from the Accident and Emergency (A&E) Department; 3 by general practitioners (GP) and 2 were referred by their dentists. Table 1 (Tab. 1) summarizes the study characteristics.

One patient had swallowed a small piece of the denture plate and the other patient had swallowed an artificial tooth. Both patients complained about a feeling of something stuck in the throat (FOSIT), but were able to eat and drink without any struggle. Flexible pharyngolaryngoscopy did not reveal any foreign body in the upper aerodigestive tract. These patients were reassured and discharged home the same day. No long-term problems were reported.

**Figure 1:**
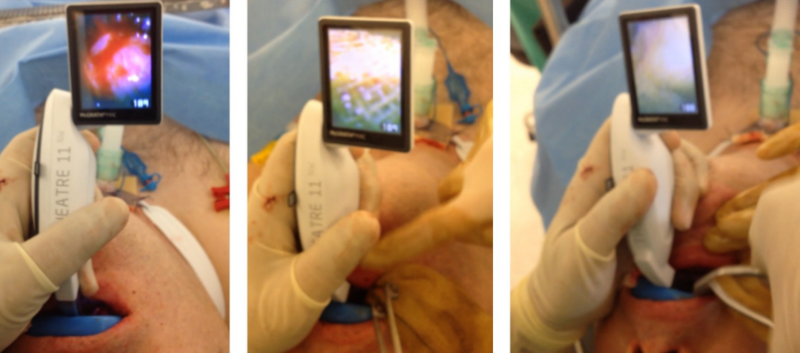
An example of video laryngoscope and Magill forceps in use

Two patients were referred to us by their dentists after these patients swallowed a part of the denture plate. Both patients complained about pain in the throat along with odynophagia and dysphagia. One patient was noticed to have a 1 cm piece of denture material, which was successfully removed in the clinic without any sedation. The other patient was taken to the theatre, and a 1.5 cm piece of denture plate was removed under sedation using Magill forceps and video laryngoscope (Figure 1 (Fig. 1)). No post-operative complications were encountered.

**Figure 2:**
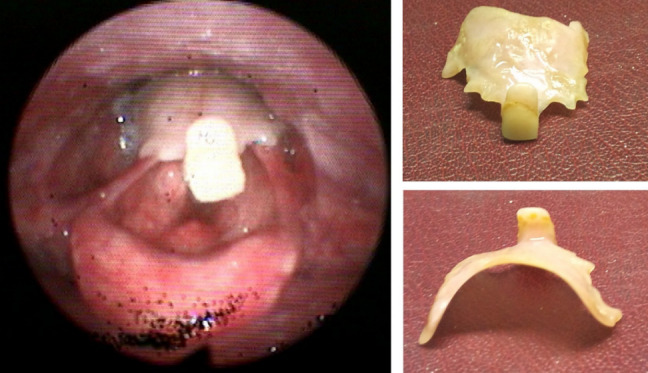
Flexible laryngoscopy: the denture can be seen covering the posterior part of the larynx. The patient did not have any respiratory distress but complained about absolute dysphagia and pain in throat.

Six patients presented with accidental swallowing of the denture plate. They complained about absolute dysphagia and pain in the throat worse on swallowing. There was no respiratory distress or drooling. The presence of denture in the hypopharynx was confirmed on flexible pharyngolaryngoscopy (Figure 2 (Fig. 2)). The patients were taken to the theatre, and under light sedation the dentures were removed by Magill forceps without any complications. In most situations, the anaesthetist was able to remove the denture plate without any difficulty under intubating laryngoscope guidance using Magill forceps. Four patients went home the same day while three patients stayed overnight.

**Figure 3:**
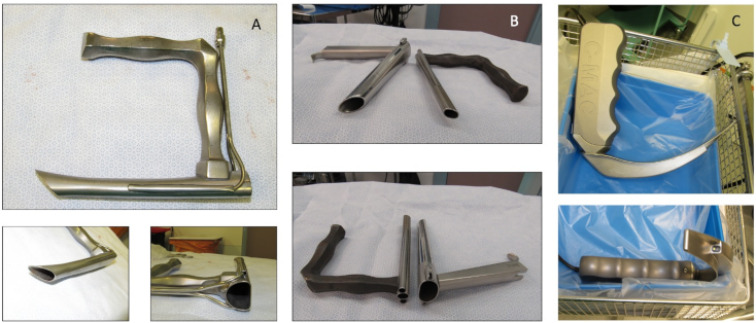
Rigid laryngoscopes; curved and straight laryngoscopes (A, B) and video laryngoscope (C)

**Figure 4:**
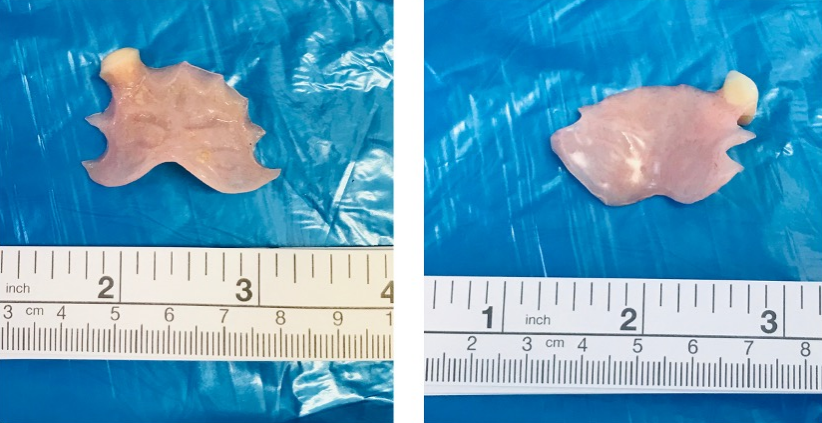
Denture plate with sharp edges

**Figure 5:**
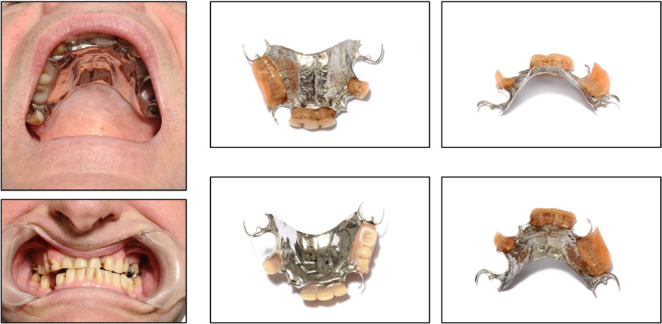
Metallic denture with sharp pointed hooks and edges

The remaining 24 patients required general anaesthesia to deal with the swallowed denture. Out of 24 patients, 19 underwent rigid pharyngo-oesophagoscopy using rigid laryngoscope, video laryngoscope, pharyngoscope and short oesophagoscope (Figure 3 (Fig. 3)). The denture was removed successfully without any complications (Figure 4 (Fig. 4)). In these patients, the denture was impacted at the level of the cricopharyngeus muscle or upper oesophagus. Awake fibreoptic intubation was used in a patient with partial blockage of the supraglottis by the metallic denture with sharp edges embedded in the pharyngeal walls (Figure 5 (Fig. 5)). All these patients were discharged home within 24 hours after admission.

The remaining 5 patients had a complicated course with their swallowed dentures. Their management is described in detail in the following.

**Figure 6:**
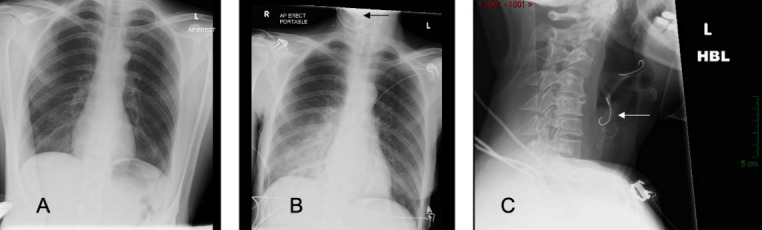
The X-rays in a patient who swallowed her denture with metal wires. On first attendance, the chest X-ray was clear (A); Chest X-ray on second admission included part of her lower neck which revealed denture wires in her pharynx (B), confirmed on soft tissue neck X-ray (C).

**Figure 7:**
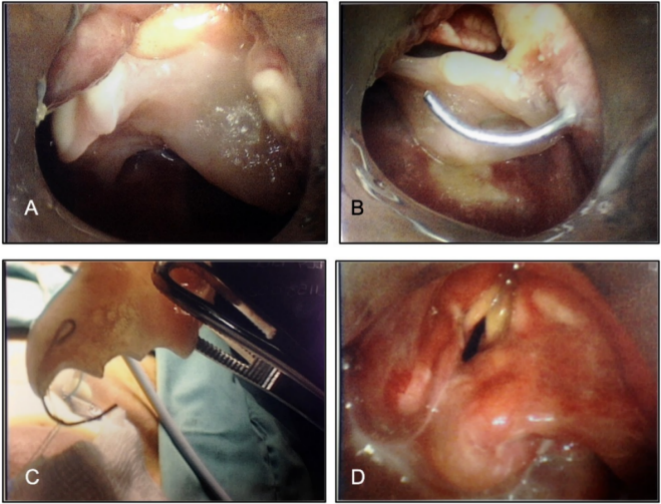
Rigid pharyngo-laryngoscopy in a patient with complete blockage of the larynx with denture. The denture plate completely covering the supraglottis with no view of the vocal cords (A). Gentle mobilisation with laryngoscope allowed disengagement of the denture wires from the pharyngeal wall (B). The denture plate removed with Magill forceps (C). Generalised erythema of the supraglottis and oedema of the right arytenoid visible after denture removal (D).

A 54-year-old Caucasian female with multiple sclerosis attended the local A&E Department after losing her denture. She was not sure if she had swallowed her denture plate, but complained about pain in her throat and dysphagia. She underwent X-rays of her chest and abdomen, which revealed no foreign body. The patient was reassured and discharged home (Figure 6 (Fig. 6)). The following morning, she re-attended the A&E Department with her husband who was her main carer. She was in respiratory distress with reduced oxygen saturation on room air. An X-ray of her neck showed a denture plate with wires (Figure 6 (Fig. 6)). The denture plate was seen on flexible laryngoscopy covering the whole supraglottis with no view of the vocal cords. The wires of the denture were embedded in the pharyngeal mucosa. The patient was transferred to the theatre and a tracheostomy was carried out under local anaesthetic to secure the airway. The denture plate was removed in one piece using Magill forceps and rigid laryngoscope (Figure 7 (Fig. 7)). The patient made an uncomplicated recovery and the tracheostomy tube was removed after 72 hours. The patient was discharged home within 48 hours afterward.

**Figure 8:**
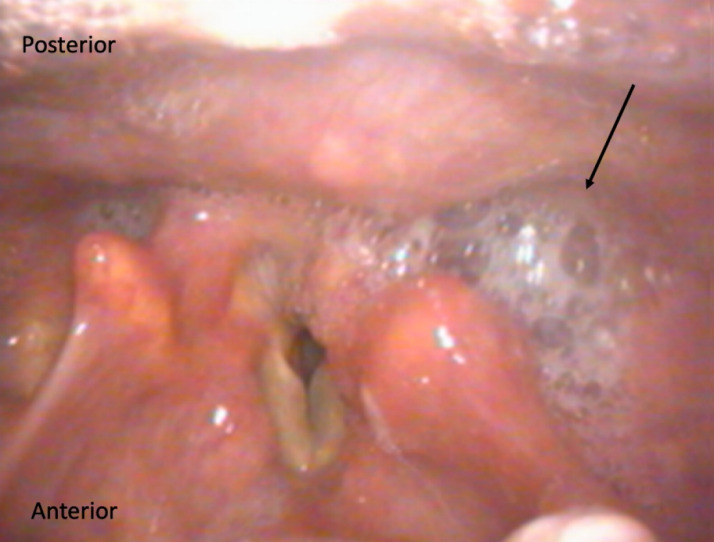
An example of pooling of saliva in the hypopharynx, worse on the left side as noticed on flexible laryngoscopy suggestive of some obstructive pathology in the hypopharynx. Such patients should be considered for examination under anaesthetic.

A 61-year-old Caucasian male woke up from sleep choking after swallowing his partial denture plate. He stated always sleeping wearing his denture plate and using CPAP machine for his sleep apnoea. The flexible laryngoscopy showed some pooling of saliva in the hypopharynx, but did not show any foreign body (Figure 8 (Fig. 8)). The neck and chest X-rays revealed wires of the swallowed denture in his upper oesophagus. The rigid oesophagoscopy showed denture plate impacted in the mid-oesophagus with wires embedded in the posterior oesophageal wall distally. The procedure was abandoned. The following morning, the patient underwent upper GI endoscopy when the denture plate was pushed into the stomach under direct vision. Diagnostic laparoscopy was then performed, a diathermy cut made in the anterior wall of the stomach, and simultaneous OGD carried out to push the denture through the gastrostomy and placed in an extraction pouch. The infra-umbilical incision was enlarged to facilitate safe extraction of the denture. The patient made a full uncomplicated recovery and was discharged home within 48 hours after the procedure.

One patient suffered from oesophageal perforation by the impacted denture in the hypopharynx. The patient was kept nil by mouth after trans-oral extraction of the denture plate. There was no obvious perforation noticed on pharyngo-oesophagoscopy, but a nasogastric feeding was inserted under direction vision in the theatre after denture removal. The patient was managed conservatively with spontaneous healing of the perforation. The patient stayed in the hospital for 21 days.

Two patients underwent neck exploration and removal of the impacted denture in the upper oesophagus. In both of these patients, it was not possible to extract the impacted denture through the mouth.

**Figure 9:**
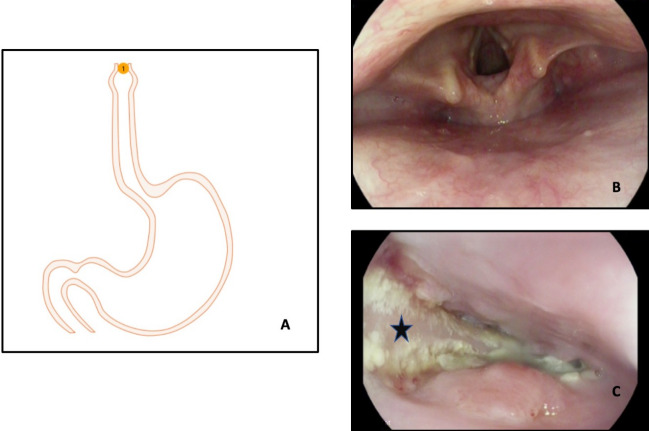
Flexible upper gastrointestinal endoscopy showing absence of any foreign body in the pharynx (B), and impacted denture was found in the upper oesophagus (A and C).

**Figure 10:**
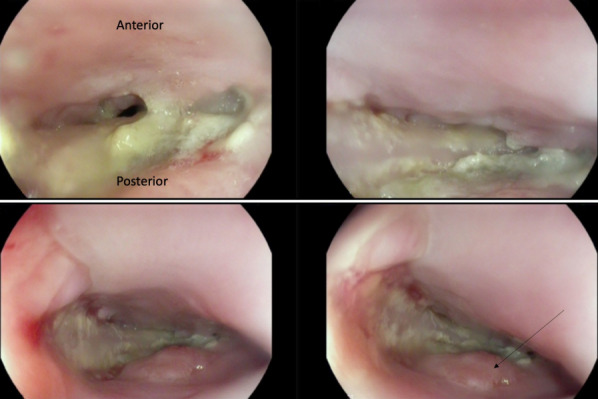
It was not possible to pass the scope beyond the impacted denture which was covered with a lot of inflammatory granulation tissue (arrow).

One of these patients was a 52-year-old Caucasian male who choked on his partial denture plate and swallowed it. An X-ray at that time did not show any foreign body. The patient continued to experience dysphagia and was referred for upper gastrointestinal endoscopy (UGIE) by his GP. He was found to have an impacted denture plate in his upper oesophagus on UGIE (Figure 9 (Fig. 9), Figure 10 (Fig. 10)). At 9 weeks since he swallowed his denture, he underwent rigid oesophagoscopy, but the denture could not be retrieved trans-orally. Left neck skin crease incision was made and dissection carried out to identify the anterior border of the sternocleidomastoid muscle. The denture could be palpated in the upper oesophagus which was incised linearly to allow extraction of the denture in one piece. The oesophagotomy was closed in two layers. The patient was kept nil by mouth and fed through the NG tube. The contrast swallow at day 4 did not show any oesophageal perforation when oral feeding was resumed. The patient made an uneventful recovery and was discharged home on day 8 after admission.

The second patient was a 57-year-old Caucasian male who choked on his denture. There was an oesophageal perforation on attempted removal of his swallowed denture through the mouth. The denture was eventually removed through the neck via external approach. The patient stayed in the hospital for 43 days but made an excellent recovery without any long-term problems.

## Discussion

Both partial and complete dentures are at risk of being swallowed, particularly if these are ill-fitting. The broken dentures can have sharp edges, or there may be sharp wires attached to the dentures, making them extremely dangerous foreign bodies in the upper aerodigestive tract. Swallowed dentures can pose some significant risks to patients. Therefore, an effective management of swallowed dentures is essential to ensure the safety of patients.

The mean age of patients with swallowed dentures from this study was 60 years. This shows that the older population are more at risk of swallowing dentures, which is to be expected, given that the older population are more likely to be edentulous. The decreased sensation in the oral cavity and decreased motor function of the laryngopharynx increase the risk of the elderly swallowing dentures [10]. In our case series, one patient suffered from multiple sclerosis and was noticed to have reduced pharyngeal sensations. Multiple co-morbidities could also potentially lead to this event, as impaired cognitive function or consciousness may well be contributing to a lack of awareness; hence, some swallowed dentures may be missed until patients present with complications [11]. One of our patients had an ingested denture impacted in his upper oesophagus for 4 months before it was finally identified and removed.

From this study, the majority of patients with swallowed dentures were predominantly male (71%) compared to female (29%). Within the general population, there is a reported majority of females wearing dentures as opposed to males [12], [13]. Most patients presented to the A&E Department and needed acute admission. This is particularly important as earlier presentation, detection and prompt removal of the denture from the upper aerodigestive tract would seem logical. Some of the patients in our cohort had a delayed presentation, leading to significant embedding of the swallowed denture and associated complications. Two of our patients required extraction of the dentures through an external approach via neck exploration.

Patients with swallowed dentures can present with a variety of symptoms from mild FOSIT or dysphagia to life-threatening acute airway obstruction, perforation or sepsis from aspiration [14]. One case report describes a large haemorrhage resulting from vascular erosion of a missed impacted denture [15]. Another case report describes a patient presenting with symptoms of dysphagia, dysphonia and weight loss, resulting in a bilateral vocal cord palsy. Malignancy was suspected from the history, examination and imaging, but impacted denture was only diagnosed during examination under anaesthesia [16]. This highlights the importance of prompt management of swallowed foreign bodies to minimise these complications. Dysphagia was the main symptom described by most people in this study. Another study also described dysphagia as a main symptom, but other symptoms included pain, odynophagia and discomfort in the neck [17].

Chest radiographs play an important role in the management of swallowed dentures. In this study, 91% of patients had a chest radiograph taken. Though X-rays are essential to assess for any damage that may have occurred as a result of the dentures, including pneumomediastinum and surgical emphysema, it has been suggested that they are less effective in detecting the presence of a foreign body as the majority of dentures are not radio-opaque [16]. They can help to guide further investigations or prompt treatment including abdominal radiographs and endoscopy [18]. We would agree that chest X-ray should be carried out, but would strongly recommend including neck in the X-ray. In one of our patients, the X-ray of chest and abdomen failed to raise any concern about the presence of denture in the pharynx. It was clearly visible on an X-ray of her neck, which was carried out when she re-attended the A&E with worsening throat pain, dysphagia and respiratory distress (Figure 6 (Fig. 6)). One study suggests that radiographs may be useful, but CT scan should be the gold standard to prevent missed dentures [19]. In one of our patients, the dysphagia was thought to be secondary to possible hypopharyngeal malignancy, when in reality it was an impacted partial denture in the hypopharynx which was not picked up on the CT scan [16]. Maybe in select cases contrast swallow should be considered to identify any impacted foreign body in the oesophagus.

Management of swallowed dentures involves a multi-disciplinary team approach depending on location and severity of the problem [17]. Management of swallowed dentures has been discussed by Gachabayov et al. [11] and includes three different approaches: observation, endoscopy and surgery. Okugbo et al. and Orji et al. support the use of oesophagoscope as the main approach for oesophageal dentures and go for open surgery if needed [17], [20]. Calder and McGuinness, and Dalvi et al. describe the technique of splitting the denture plate and removing it in pieces, however problems can be encountered with this method when the plate is too hard to cut through [21], [22]. Kuo et al. highlight the risk of complications such as airway obstruction, laryngeal spasms and oedema as a result of multiple attempts at removing the foreign body [23]. Endoscopic removal of dentures carries a significant risk of perforation due to the attached wiring and the irregular shape of the foreign body. This risk is further increased if the denture was swallowed some time ago and there is surrounding inflammation at the site of impaction [24]. One patient in our study suffered oesophageal perforation following attempted endoscopic denture removal. It has been suggested that operative extraction of ingested dentures is the safest management, in particular, an open oesophagotomy [24]. Other operative approaches described in the literature are cervical oesophagotomy, thoracotomy and thoracoscopic removal [25], [26], [27]. Twenty-four patients in this study had denture removal in the theatre, of which 2 patients required additional surgical intervention in the form of tracheostomy and gastrostomy, highlighting the complications that can be associated with swallowed dentures.

Education is vital in preventing denture aspiration so patients are more aware of their complications and understand the importance of follow-up review and care of dentures [17], [28], [29]. Therefore it is important to cultivate this positive culture to ensure continuity of care of patients with dentures whilst reducing the rate of complications. Recognising a missing denture could significantly help the situation as this prompts further investigations including laryngoscopy, X-rays, contrast swallow and CT scan in select cases of dysphagia. This is apparent in a study by Bandhopadhyay et al. [29] in which a majority of patients presented with a history of swallowed denture followed by other symptoms like difficulty swallowing and sensitivity of trachea; although is it also discussed that this may not always be apparent, depending on various patient factors including cognition status and intoxication.

The management algorithm for ingested foreign body suggested by Kuo et al. [23] is helpful and should be considered when dealing with swallowed denture. However, based on our experience, following are some of the scenarios of accidental denture swallowing and recommended management:

1. The patient swallowed one or more artificial teeth and complains of feeling of something stuck in the throat with mild throat discomfort. The flexible laryngoscopy does not show any foreign body and there is no pooling of saliva in the hypopharynx. The patient is eating and drinking without any difficulty. Reassure and discharge the patient with an advice to return if he experiences worsening of symptoms.
2. The patient swallowed part of the denture which is confirmed on laryngoscopy. The partial denture is occupying the hypopharynx and there is no obstruction to the larynx. The patient is able to breathe and speak without any difficulty. The patient should be taken to the theatre and the denture plate should be removed under light sedation using Magill’s forceps and rigid laryngoscope.
3. The patient swallowed the denture, and laryngoscopy confirms it lying in the hypopharynx with partial blockage of the supraglottis. The patient should receive awake fibreoptic nasal or oral intubation in the theatre with subsequent trans-oral removal of the denture plate.
4. The patient swallowed the denture, and laryngoscopy confirms it covering the whole of the larynx with no view of the supraglottis and glottis. The patient needs an urgent tracheostomy under local anaesthetic to secure the airway. The patient should then undergo direct pharyngolaryngoscopy and removal of the denture plate.
5. The patient swallowed the denture, but there is no foreign body seen in the hypopharynx. The patient is complaining of dysphagia with or without odynophagia. The patient needs general anaesthetic and trans-oral intubation with a standard endotracheal tube. The patient needs examination with rigid laryngoscope, hypo-pharyngoscope or short oesophagoscope to locate the denture plate. If possible, the denture would be removed trans-orally using a heavy grasping forceps. If not possible and the denture is impacted in the upper part of the oesophagus, then it is better to remove it through the neck via external approach. If the denture is located in the mid-oesophagus and it is not possible to retrieve it through the mouth, then it would be best pushed into the stomach under direct vision followed by laparoscopy and gastrostomy approach extraction.

## Conclusions

Swallowed dentures can pose a significant diagnostic and therapeutic challenge. In the absence of a clear evidence of denture ingestion, a detailed history and examination are needed to identify this serious pathology. The diagnosis can be challenging, particularly due to the radiolucent nature of dentures. Sharp metals, i.e. clasps, especially on removable partial dentures (RPD) can cause serious injuries. Once confirmed, the ingested denture should be removed as soon as possible to minimize the risk of serious complications. A multidisciplinary team approach is recommended when dealing with an impacted swallowed denture including the ENT surgeon, upper GI surgeon, gastroenterologist, and an experienced anaesthetist.

## Notes

### Informed consent

Informed consent has been obtained from the patients for the publication of this case report.

### Prior presentation

Poster presentation at the ASiT International Conference, 28th–30th March 2014, Belfast, UK.

### Competing interests

The authors declare that they have no competing interests.

## Figures and Tables

**Table 1:**
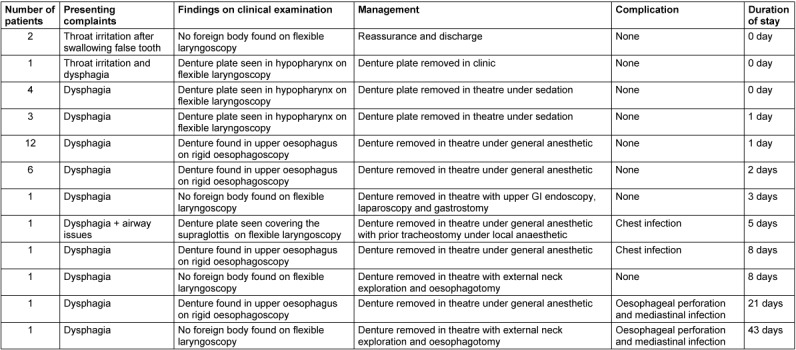
Study characteristics
